# Cognitive impairment in metabolically-obese, normal-weight rats: identification of early biomarkers in peripheral blood mononuclear cells

**DOI:** 10.1186/s13024-018-0246-8

**Published:** 2018-03-22

**Authors:** Margalida Cifre, Andreu Palou, Paula Oliver

**Affiliations:** Laboratory of Molecular Biology, Nutrition and Biotechnology, Universitat de les Illes Balears and CIBER de Fisiopatología de la Obesidad y Nutrición (CIBERobn), Cra. Valldemossa Km 7.5, E-07122 Palma de Mallorca, Spain

**Keywords:** MONW, MCI, Blood cells, Hippocampus

## Abstract

**Background:**

Metabolically-obese, normal-weight (MONW) individuals are not obese in terms of weight and height but have a number of obesity-related features (e.g. greater visceral adiposity, insulin resistance, and increased risk of cardiovascular disease). The MONW phenotype is related to the intake of unbalanced diets, such as those rich in fat. Increasing evidence shows a relationship between high-fat diet consumption and mild cognitive impairment and dementia. Thus, MONW individuals could be at a greater risk of cognitive dysfunction. We aimed to evaluate whether MONW-like animals present gene expression alterations in the hippocampus associated with an increased risk of cognitive impairment, and to identify early biomarkers of cognitive dysfunction in peripheral blood mononuclear cells (PBMC).

**Methods:**

Wistar rats were chronically fed with a 60% (HF60) or a 45% (HF45) high-fat diet administered isocalorically to control animals to mimic MONW features. Expression analysis of cognitive decline-related genes was performed using RT-qPCR, and working memory was assessed using a T-maze.

**Results:**

High-fat diet consumption altered the pattern of gene expression in the hippocampus, clearly pointing to cognitive decline, which was accompanied by a worse performance in the T-maze in HF60 animals. Remarkably, *Syn1* and *Sorl1* mRNA showed the same expression pattern in both the hippocampus and the PBMC obtained at different time-points in the HF60 group, even before other pathological signs were observed.

**Conclusions:**

Our results demonstrate that long-term intake of high-fat diets, even in the absence of obesity, leads to cognitive disruption that is reflected in PBMC transcriptome. Therefore, PBMC are revealed as a plausible, minimally-invasive source of early biomarkers of cognitive impairment associated with increased fat intake.

**Electronic supplementary material:**

The online version of this article (10.1186/s13024-018-0246-8) contains supplementary material, which is available to authorized users.

## Background

Dementia is a general term used to define ageing-associated cognitive disorders and includes diseases that affect mental abilities severely enough that the ability to perform daily tasks is impaired. Among these disorders, Alzheimer’s disease (AD) and vascular dementia are included [1]. Some authors suggest that there is a transition phase referred to as mild cognitive impairment (MCI) in which individuals do not yet meet the criteria for dementia, but are at a greater risk of developing it [2, 3]. The risk of MCI increases with age, and men appear to be at higher risk than women [3]. Moreover, several lines of evidence show that obesity in mid-life is a risk factor not only of MCI at old age, but additionally, late-life dementia and AD [4–7]. Moreover, a growing body of research points towards diet playing a possible role in modulating the risk of cognitive decline and dementia [8, 9]. A macronutrient manifested as an important factor is dietary fat [9, 10], since most studies show that there is increased risk of cognitive dysfunction or late-life dementia with higher intakes of dietary fat, particularly saturated fatty acids [10–12].

The intake of fat-rich food is steadily increasing in Western societies [13]. It is well known that dietary fat intake is positively correlated with the development of obesity and the incidence of its comorbidities (such as insulin resistance, cardiovascular disease (CVD) and/or cancer) [14, 15]. Furthermore, associations between obesity and impaired cognitive function have more recently been recognised [16]. However, the negative effects of an unbalanced diet on the disruption of metabolism are not necessarily correlated with obesity. In fact, there is a group of subjects who present metabolic features related to obesity, but in the absence of increased body weight. These individuals are known as “metabolically-obese, normal-weight” (MONW), and have greater visceral adiposity and increased fat deposition in tissues such as liver, heart, and muscle [17–19], which can lead to hyperinsulinemia, insulin resistance, type 2 diabetes mellitus, and CVD [20, 21]. MONW subjects have recently been reported to represent around 20% of the world population, with greater prevalence among men (25% for men vs 19% for women) [22]; this could be problematic, as it would mean that a significant part of the population could be at high metabolic risk, but most of them are not being diagnosed because they are not overweight or obese. The intake of diets with an unbalanced macronutrient proportion (rich in fats or simple carbohydrates) is one of the main causes of the increasing emergence of MONW subjects [23, 24]. As stated earlier, a high-fat diet appears to play a significant role in cognitive dysfunction and, thus, MONW individuals could be at a higher risk of developing cognitive impairment later in life.

How obesity or high-fat diet intake affect brain physiology and function remains poorly understood, but these conditions are known to have a great impact on the hippocampus [25–27]. The hippocampus is a vital structure for cognition; it has a significant role in memory and learning, and is particularly vulnerable to ageing [28, 29]. Since the diagnosis of MCI or dementia would require a number of established clinical features that are present when the disorder is almost or fully developed, and given the complication of obtaining samples of brain tissue, the need for readily available early biomarkers of poorly invasive samples has proven to be urgent. In this sense, peripheral blood mononuclear cells (PBMC) could be a very interesting biological material. The gene expression pattern of these cells is increasingly used for diagnosis, to predict the clinical outcome of various diseases [30, 31], and in nutrigenomic studies [32–34]. Moreover, PBMC are easily obtained, offering the chance to perform studies at different time points. In this way, it would be possible to find early biomarkers of disease and, thereby, contribute to setting and addressing strategies to prevent or delay the onset and/or progression of dementia.

Here, a moderately high-fat (45% kcal from fat, HF45) or a very high-fat (60% kcal from fat, HF60) diet was administered in isocaloric conditions to a control diet in order to avoid overweight and to mimic the situation of “normal-weight obesity” in Wistar rats. We aimed to evaluate whether MONW-like animals present gene expression alterations in the hippocampus associated with an increased risk of cognitive impairment. Moreover, we aimed to determine whether these changes are reflected in PBMC in order to identify early biomarkers of cognitive dysfunction in blood samples.

## Methods

### Animals, diets and experimental design

Two-month old male Wistar rats (Charles River Laboratories España SA, Barcelona, Spain), were housed in plastic cages (one rat per cage) at 22 °C with a 12 h light/12 h dark cycle. Male animals were used to avoid potential interferences due to hormonal fluctuations in females. Moreover, in a number of animal studies, our group has demonstrated clear sexual dimorphism in various functions related to metabolism that make females more protected from obesity-related disorders [35, 36]. After acclimatization for 7 days on a normolipidic control balanced diet, rats were randomly assigned to three groups for a four-month dietary intervention: control group (*n* = 10), HF45 diet group (*n* = 10), and HF60 diet group (*n* = 10). The control group was fed a normolipidic diet (D12450B, Research Diets) containing 70% of energy (kcal) from carbohydrates, 10% from fats, and 20% from proteins. The HF45 group was fed a hyperlipidic diet (D12451, Research Diets) containing 35% of energy from carbohydrates, 45% from fats, and 20% from proteins. HF60 animals were fed a hyperlipidic diet (D12492, Research Diets) containing 20% of energy from carbohydrates, 60% from fats, and 20% from proteins. All diets were purchased from Brogaarden (Gentofte, Denmark). During the whole intervention period, diets were administered to all experimental groups in isocaloric conditions, relative to the control group. To calculate energy (kcal) consumption in the control group, animals in this group had free access to food and their food intake was recorded daily. Animals in the HF45 and HF60 groups received an amount of food containing kcal equal to the average amount consumed by the control group the day before. The energy density of the diets used for the calculations was 3.85, 4.73, and 5.24 kcal/g, for the control, HF45, and HF60 diets, respectively. Food intake of all groups was recorded daily to calculate daily caloric intake and cumulative caloric intake throughout the experiment; body weight was recorded three times a week.

At the beginning of the experiment and at the age of three, five, and six months, blood samples (1.5–2 ml) were collected in fed conditions from the saphena vein using EDTA 100 mM as an anticoagulant. PBMC fraction was isolated by Optiprep™ (Sigma Aldrich Química SA, Madrid, Spain) density gradient separation according to the manufacturer’s instructions. At the same time, blood samples from the saphena vein were harvested without an anticoagulant, stored for 1 h at room temperature, and then centrifuged at 1000 g for 10 min at 4 °C to collect serum samples. In addition, animals were subjected monthly to 12–14 h nocturnal fasting for serum collection in order to determine the homeostatic model assessment for insulin resistance (HOMA-IR) index using the formula described by Matthews et al. [37]: fasting serum insulin levels (mU/l) per fasting serum glucose (mmol/l)/ 22.5.

At six months of age, animals were sacrificed by use of a guillotine under ad libitum feeding conditions. The left hippocampus was collected and immediately frozen in liquid nitrogen and stored at − 80 °C for gene expression analysis, while the right hippocampus was fixed by immersion in 4% paraformaldehyde in 0.1 M sodium phosphate for future histological analysis. Different white adipose tissue depots (epididymal, inguinal, mesenteric, and retroperitoneal) were removed and weighed to determine the adiposity index, and then immediately frozen in liquid nitrogen and stored at − 80 °C. Additionally, truncal blood was collected from the neck to collect serum.

### Adiposity

Adiposity was determined by an adiposity index computed for each rat as the sum of epididymal, inguinal, mesenteric, and retroperitoneal white adipose tissue depot weights, and expressed as a percentage of total body weight. In addition, body composition was measured monthly using an EchoMRI-700™ (Echo Medical Systems, LLC., TX, USA) without anaesthesia. Direct measurements of fat and lean mass (in grams) were taken using the analyser, and expressed as a percentage of total body weight.

### Fat liver content

Total lipid levels were determined in the liver using the Folch method, which uses chloroform and methanol as solvents to extract lipids [38]. Briefly, liver tissue (0.4 g approximately) was homogenised with 5 ml of chloroform:methanol (2:1). After washing steps with sodium chloride and centrifugations, a lipid extract was obtained which was dispensed in pre-weighted Folch vials. The vials were heated up to facilitate chloroform evaporation, and lipid content was determined by weighing each Folch vial.

### Quantification of circulating insulin and glucose levels

Serum insulin levels were measured using a rat insulin enzyme-linked immunosorbent assay (ELISA) kit (Mercodia AB, Uppsala, Sweden), and blood glucose was tested using an Accu-Chek Glucometer (Roche Diagnostics, Barcelona, Spain). Both variables were measured in feeding and fasting conditions.

### Total RNA isolation

Total RNA from the hippocampus and PBMC samples was isolated using Tripure reagent (Roche Diagnostics Barcelona, Spain) following the manufacturer’s protocol. RNA yield was quantified on a NanoDrop ND 1000 spectrophotometer (NanoDrop Technologies, Wilmington, DE, USA), and RNA integrity and purity was confirmed using 1% agarose gel electrophoresis.

### Reverse transcription quantitative real-time polymerase chain reaction (RT-qPCR) analysis

The expression of different genes involved in metabolic processes related to cognition (see Table 1) were analysed by RT-qPCR in the hippocampus, and selected genes also in PBMC. For the hippocampus, 0.1 μg of total RNA (in a final volume of 5 μl) was denatured at 65 °C for 10 min and then reverse transcribed to cDNA using MuLV reverse transcriptase (Applied Biosystem, Madrid, Spain) at 20 °C for 15 min, 42 °C for 30 min, and with a final step of 5 min at 95 °C. For PBMC, equal amounts of total RNA (0.05 μg) were reverse transcribed into cDNA using an iScript™ cDNA synthesis kit (Bio-Rad Laboratories, Madrid, Spain). Both reactions were carried out in an Applied Biosystems 2720 Thermal Cycler (Applied Biosystem, Madrid, Spain). After cDNA synthesis, real-time PCR was performed to determine mRNA expression of cognitive impairment-related genes. Each PCR was carried out from diluted (1/10 for hippocampus, 1/5 for PBMC) cDNA template, forward and reverse primers (5 μM) and Power Sybr Green PCR Master Mix (Applied Biosystems, Madrid, Spain), following previously described reaction conditions [33]. The threshold cycle (Ct) was calculated by the instrument’s software (StepOne Software v2.0, from Applied Biosystems), and the relative expression of each mRNA was calculated as a percentage of control rats, using the 2^−ΔΔCt^ method [39]. Data from the hippocampus was normalized against two reference genes (*Gdi* and *Rplp0*). For PBMC, results were normalized against integrin (*Itgβ1*). Primers for different genes are shown in Additional file 1. *Gdi* and *Itg1β* were used since they have been previously described as good constitutive genes based on microarray studies [40, 41], while *Rplp0* has been previously described as a useful housekeeping gene [42]*.* All primers were obtained from Sigma Genosys (Sigma Aldrich Química SA, Madrid, Spain).

**Table 1:** Cognitive-related genes analysed in the study, divided into the metabolic processes they are involved in

Process	Gene symbol	Gene name
Amyloid peptide metabolism	*App*	Amyloid precursor protein
	*Naat16*	N(alpha)-acetyltransferase 16, NatA auxiliary subunit
	*Sorl1*	Sortilin related receptor 1
	*Tmcc2*	Transmembrane and coiled-coil domain family 2
Neuronal viability/function	*Bdnf*	Brain derived neurotrophic factor
	*Casp3*	Caspase 3
	*Fndc5*	Fibronectin type III domain containing 5
Transcription factor-mediated regulation of cognitive-related processes	*Creb*	cAMP responsive element binding protein 1
	*Nrf2*	NF-E2-related factor 2
	*Pgc1α*	PPARG coactivator 1 alpha
	*Zpr1*	Zinc finger protein 259
Synaptic function/signalling	*Syn1*	Synapsin I
	*Trkb*	Neurotrophic receptor tyrosine kinase 2
Inflammation	*Tnf α*	Tumour necrosis factor α

### Western blot analysis

Three selected proteins, sortilin, synapsin I, and TMCC2 were analysed by Western blot in the hippocampus using 15 μg of total protein per lane in a 4–20% Criterion TGX Precast Gel (Bio-Rad, Madrid, Spain). Membranes were incubated overnight with rabbit polyclonal anti-Sortilin (catalogue number: S0697; Sigma-Aldrich, Madrid, Spain), anti-Synapsin I (ab64581; Abcam, Madrid, Spain), or anti-TMCC2 (25042–1-AP; Proteintech, AntibodyBcn, Barcelona, Spain) antibody. Antibodies were diluted 1:400, 1:1000, and 1:1000, respectively, in 0.02 M Tris-buffered saline and 0.1% Tween-20. Beta actin antibody (37,005, Cell Signalling, Werfen, Barcelona, Spain) was used as transfer and loading control. Infrared-dyed secondary anti-IgG antibodies (LI-COR Biosciences, Lincoln, NE, USA) were used, membranes were scanned in Odyssey Imager (LI-COR Biosciences).

### Immunohistochemistry analysis of GFAP in the hippocampus

Five-micrometre-thick longitudinal sections of the hippocampus containing the whole microglia (one section per animal from the different experimental groups) were immunostained by means of the avidin-biotin technique [43]. Briefly, sections were pre-treated with citrate buffer pH 6 for 20 min in a microwave oven at maximum power followed by incubation with normal goat serum. Then, they were incubated overnight at 4 °C with primary rabbit polyclonal anti-GFAP antibody (Abcam plc, Cambridge, UK) diluted 1:400 with PBS. Sections were then incubated with goat anti-rabbit IgG secondary antibody (Vector Laboratories, Burlingame, CA, USA) diluted 1:200, and afterwards with ABC complex (Vectastain ABC Kit, Vector Laboratories). Peroxidase activity was revealed with Sigma Fast 3,3′- diaminobenzidine (Sigma-Aldrich, St Louis, MO, USA) as a substrate. Finally, sections were counterstained with haematoxylin and mounted in Eukitt (Kindler, Germany). Images were acquired with a Zeiss Axioskop 2 microscope equipped with an AxioCam ICc3 digital camera and AxioVision 40 V 4.6.3.0 Software (Carl Zeiss, S.A., Barcelona, Spain). For the morphometric study, the number of GFAP-stained astrocytes from the whole glia present in the hippocampus samples were counted.

### Behavioural testing: T-maze alternation

The effect of hyperlipidic diets on cognitive function was assessed using a spontaneous alternation paradigm in a T-maze, following a protocol from Deacon & Rawlins [44]. Spontaneous alternation is a short-term memory task that assesses the ability of an animal to remember the arm in a T-maze that it had previously entered, and to select an alternative maze arm when re-exposed to the apparatus. At six months of age, rats (*n* = 7 per group) were tested using a free-trial procedure. Briefly, each animal was placed at the start area of the T-maze and allowed to choose an arm to enter. Once the rat had selected an arm, it was confined to that arm for 30 s and then returned to the start arm and confined there for 10 s. Afterwards, the rat was allowed to choose an arm again. Five trials were performed per rat. Each animal received a score of 0 if it chose the same arm, and a score of 1 if it alternated arms, and a corrected percentage of success per animal was calculated. Further, latency (time taken by the animals to select the arm) was recorded to detect potential delays in the response and, therefore, rule out possible brain dysfunction which could slow down decision processes.

### Statistical analysis

All data are shown as mean ± SD. Normal distribution of the data and homogeneity of variances were tested using the Shapiro-Wilk test and the Levene test, respectively. Statistical significance of the HF45 or HF60 vs control was also analysed using an unpaired *t*-test. Additionally, one-way ANOVA followed by an LSD post hoc test was used for multiple comparisons. A Pearson correlation between anthropometric and circulating parameters and gene expression was conducted. All statistical analyses were conducted with log-transformed variables if they were not adjusted to parametric criteria. In cases where normal distribution or homogeneity of variances could not be observed, nonparametric tests were used. Statistical analyses were performed with SPSS for Windows (SPSS, Chicago, IL, USA). The threshold of significance was defined at *p* < 0.05.

## Results

### Body weight, adiposity and serum parameters

As shown in Table 2, high-fat pair-feeding did not affect body weight. Chronic exposure to a 60% HF diet caused a significant increase in fat mass content, adiposity index and a greater amount of all fat depots (subcutaneous and visceral depots). Although animals fed a 45% HF diet showed an increase in all these parameters, values did not reach statistical significance except for the inguinal fat depot. Both groups displayed greater liver-fat deposition compared to controls as well as signs of insulin resistance, such as increased fasting circulating insulin levels accompanied by alterations in glycaemia and increased HOMA-IR index.

**Table 2:** Body weight, adiposity-related parameters, serum parameters, and HOMA-IR index

		NF		HF45		HF60	
Body weight (g)		477 (27)		466 (23)		472 (32)	
Fat mass (%)		18.4 (3.3)	a	20.5 (4.8)	a,b	23.4 (4.5)	b*
Adiposity index (%)		9.32 (1.51)	a	11.2 (2.5)	a,b	13.5 (2.7)	b*
Weight of adipose tissues (g)							
eWAT		14.0 (2.1)	a	16.0 (4.5)	a,b	18.1 (5.5)	b*
iWAT		11.9 (2.5)	a	16.1 (4.6)	a,b*	15.7 (5.0)	b*
mWAT		6.89 (1.87)		7.05 (2.92)		7.90 (2.18)	
rWAT		11.9 (3.2)	a	13.6 (4.2)	a,b	17.6 (6.1)	b*
Visceral fat content (g)		32.1 (6.9)	a	36.6 (10.9)	a,b	43.6 (13.1)	b*
Liver fat content (mg/ g liver)		38.3 (6.2)	a	57.9 (15.4)	b*	56.2 (11.9)	b*
Glucose (mg·dl^−1^)	Feeding	93.3 (9.4)	a	106 (5.5)	b*	111 (13.3)	b*
	Fasting	89.6 (7.4)	a	98.4 (8.4)	b*	97.4 (7.1)	b*
Insulin (μg·l^−1^)	Feeding	2.21 (0.85)		1.88 (0.89)		1.68 (0.67)	
	Fasting	0.32 (0.11)	a	0.51 (0.28)	a,b	0.80 (0.37)	b*
HOMA-IR		1.7 (0.7)	a	2.8 (1.6)	a,b	4.5 (2.1)	b*

Data correspond to male Wistar rats fed a control diet (NF), a moderate high-fat diet (45% kcal from fats, HF45 group), or a very high-fat diet (60% kcal from fats, HF60 group) from the age of 2 months until the age of 6 months. Food in HF groups was offered in isocaloric amounts to the control group

Abbreviations: *eWAT* epididymal white adipose tissue; *iWAT* inguinal WAT; *mWAT* mesenteric WAT; *rWAT* retroperitoneal WAT

Results represent mean (SD) (*n* = 10 in all groups). Statistics: *different vs control group (Student’s *t* test, *p* < 0.05). A one-way ANOVA was performed followed by an LSD post hoc test. Values not sharing a common letter (a, b) are significantly different (*p* < 0.05). No letters = no statistical difference

### Effects of isocaloric HF diet intake on working memory

**Fig. 1:**
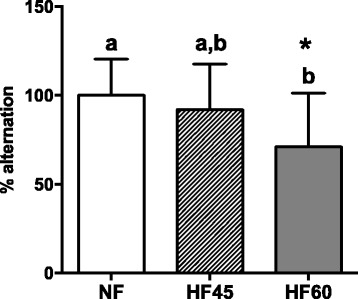
Effects of a control diet, a moderate high-fat diet (45% kcal from fats, HF45), and a very high-fat diet (60% kcal from fats, HF60) administered to male Wistar rats from the age of two months until the age of six months on working memory using spontaneous alternation paradigm in a T-maze. Food in HF groups was offered in isocaloric amounts to the NF group (control). Five trials per rat were performed and then % alternation was calculated per group. Data represent mean ± SD (*n* = 7). Statistics: * indicates that values from the HF groups are different from the control group (Student’s *t* test, *p* < 0.05). Additionally, a one-way ANOVA was performed followed by an LSD post hoc test; values not sharing a common letter (a, b) are significantly different (*p* < 0.05)

Spontaneous alternation paradigm in a T-maze capitalises on rats’ tendency to choose alternative maze arms when re-exposed to the maze. Since animals must remember the arm they have just entered to show alternation behaviour, this is a short-term memory task that is dependent on hippocampal processing [45]. As seen in Fig. 1, HF60 rats exhibited a significantly lesser preference for alternation than control animals. However, no significant differences were observed between HF45 and controls. Latencies did not differ between groups (data not shown).

### Effects of isocaloric HF diet intake on hippocampal gene expression

**Fig. 2:**
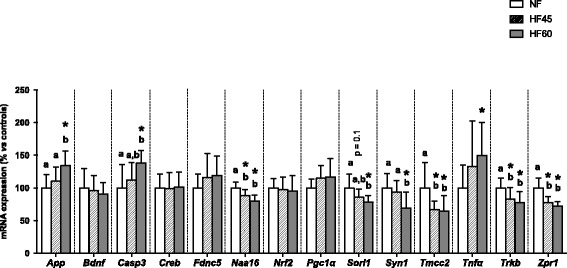
Expression of cognitive impairment-related genes in the hippocampus in the groups described in Fig. 1. mRNA expression levels were measured by RT-qPCR. Data represent mean ± SD (*n* = 10) of ratios of specific mRNA levels normalized against *Gdi1* and *Rplp0* (used as reference genes). Data of the control (NF) group were set to 100%, serving as a reference to the rest of the values. Statistics: * indicates that values from the HF groups are different from the control group (Student’s *t* test, *p* < 0.05); additionally, *p* values indicate statistical trends towards significance. Furthermore, a one-way ANOVA was performed followed by an LSD post hoc test; values not sharing a common letter (a, b) are significantly different (*p* < 0.05); no letters = no statistical difference

In the hippocampus (Fig. 2), HF60 feeding had a great impact on the expression of selected cognitive-related genes, which in most cases appeared to be dependent on the percentage of fat in the diet. Table 1 briefly summarises the metabolic processes in which the genes analysed are involved. The unbalanced diet produced a significant increase in mRNA levels of the gene coding for the amyloid precursor protein (*App*) and of proapoptotic (*Casp3*) and inflammatory (*Tnfα*) genes. A similar trend regarding the expression of these genes was observed in the HF45 group, although changes did not reach statistical significance. Levels of *Naa16*, *Sorl1*, *Tmcc2*, and *Zpr1* were significantly lower with both unbalanced diets. Moreover, the expression of *Syn1* was down-regulated with HF60 pair-feeding. No changes were observed in the expression of *Bdnf*, the gene coding for the brain-derived neurotrophic factor (BDNF), with either of the high-fat diets used. Nevertheless, the animals maintained on both high-fat diets displayed reduced expression of *Trkb*, which codes for the receptor of BDNF. No changes in mRNA levels were observed in hippocampus for the other 4 genes analysed: *Creb*, *Fndc5*, *Nrf2* and *Pgc1α*.

### Differential gene expression of cognitive impairment-related markers in PBMC during dietary treatment with isocaloric HF diets

**Fig. 3:**
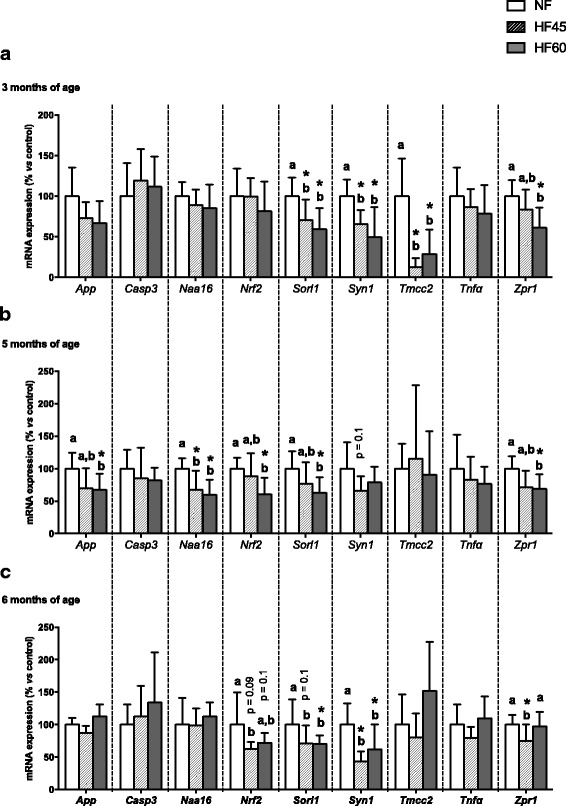
Expression of cognitive impairment-related genes in PBMC of male Wistar rats of three (**a**), five (**b**), and six (**c**) months of age submitted to a control (NF), HF45 or HF60 diet for four months. Food in the HF groups was offered in isocaloric amounts to the control group. mRNA expression levels were measured by RT-qPCR. Data represent mean ± SD (*n* = 10) of ratios of specific mRNA levels normalized against *Itg1β* (used as a reference gene). Data of the control (NF) group were set to 100%, serving as a reference to the rest of the values. Statistics: *indicates that values from the HF groups are different from the control group (Student’s *t* test, *p* < 0.05); additionally, p values indicate statistical trends towards significance. Furthermore, a one-way ANOVA was performed followed by an LSD post hoc test; values not sharing a common letter (a, b) are significantly different (*p* < 0.05); no letters = no statistical difference

After analysing the mRNA levels in the hippocampus of the above mentioned genes, the most interesting ones were selected in order to analyse their expression pattern in PBMC samples. Interestingly, PBMC were able to reflect some of the changes observed in the hippocampus. At the age of three months (after one month of nutritional intervention), changes in the expression of some genes were already evident (Fig. 3a). As observed in the hippocampus at the end of the experimental period, there was a significant decrease in *Sorl1*, *Tmcc2*, and *Zpr1* expression, which was seen in PBMC after only one month of exposure to both HF diets (Fig. 3a). Moreover, a decrease in *Syn1* expression was detected in PBMC of both HF groups even when its expression in the hippocampus was not changed in the HF45 group. At the age of five months, mRNA levels of the genes studied followed the same pattern as described at the age of three months, although statistical significance was not reached in some cases due to greater variability (Fig. 3b). Additionally, decreased expression of *App, Naa16,* and *Nrf2* was observed at this point, especially in the HF60 group; in particular, the significant reduction of *App* expression was surprising as it was increased in the hippocampus of these animals (a typical feature of AD). After four months of nutritional intervention (six months of age), *Nrf2*, *Sorl1*, and *Syn1* expression remained decreased, whereas *Zpr1* expression remained diminished only in the HF45 group (Fig. 3c). In addition to the above-mentioned markers, *Bdnf* and *Trkb* mRNA expression was also analysed in PBMC but was not detected.

### Effects of isocaloric HF diet intake on the expression of selected proteins in the hippocampus

**Fig. 4:**
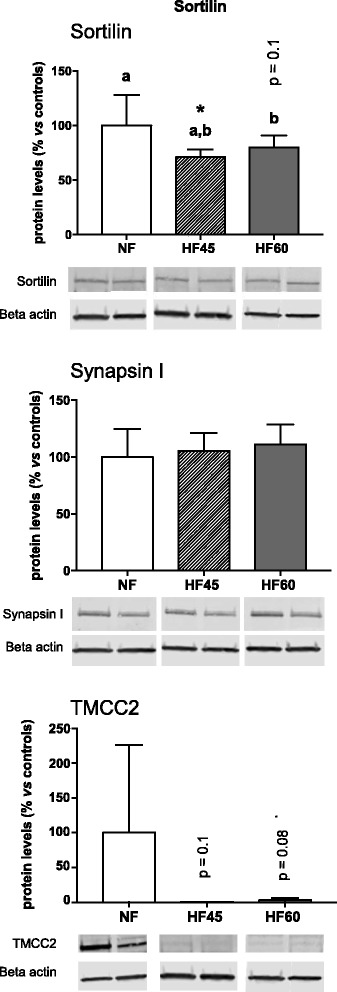
Protein levels of sortilin, synapsin I, and TMCC2 in the hippocampus in the groups described in Fig. 1 measured by Western Blot. Data represent mean ± SD (*n* = 5–7) of specific protein levels referred to beta actin (used as a reference gene). Data of the control group were set to 100%, serving as a reference to the rest of the values. Statistics: * indicates that values from the HF groups are different from the control group (Student’s *t* test, *p* < 0.05); additionally, p values indicate statistical trends towards significance. Furthermore, a one-way ANOVA was performed followed by an LSD post hoc test; values not sharing a common letter (a, b) are significantly different (*p* < 0.05); no letters = no statistical difference. Representative bands obtained in the Western Blot are shown: 15 μg of protein was loaded per lane. Beta actin was used as transfer and loading control

When comparing gene expression patterns in the HF groups, three genes, *Sorl1*, *Syn1*, and *Tmcc2*, showed a similar regulatory pattern in the hippocampus and PBMC. Thus, these genes were selected to analyse whether changes at mRNA level were correlated to changes in protein expression in the hippocampus. As observed when analysing gene expression, sortilin and TMCC2 protein levels decreased in both the HF45 and HF60 groups; however, no altered expression was found for synapsin I (Fig. 4).

### Effect of isocaloric HF diet intake on hippocampal gliosis

**Fig. 5:**
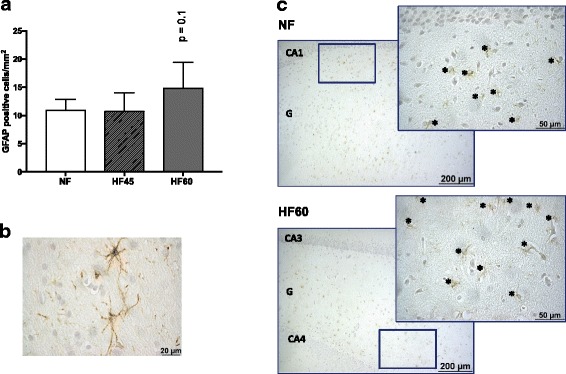
Quantification of GFAP-stained cells in the hippocampus of male Wistar rats fed a control (NF), HF45 or HF60 diet from the age of two months until the age of six months, evaluated by immunohistochemistry. Food in HF groups was offered in isocaloric amounts to the NF group (control group). **a** Number of GFAP positive cells in the NF, HF45 and HF60 groups. GFAP-stained cells were counted from the whole glia present in the hippocampus samples, and referred to the glia area (positive cells/mm^2^). Data represent mean ± SD (*n* = 6 in control and HF60 groups; *n* = 5 in HF45 group). Statistics: *P* value indicates statistical trend towards significance (Student’s *t*-test). **b** An enlarged GFAP positive cell is shown as example of the specificity of the antibody. **c** Representative hippocampus sections immunostained for GFAP in the NF and HF60 groups. Insets: enlargement of the corresponding framed areas. *indicates GFAP-positive cells. Abbreviations: CA: cornu ammonis, G: glia. Scale bar: 200 μm and 50 μm for insets

Intermediate glial fibrillary acidic protein (GFAP) was used to evaluate astrocyte hyperplasia [46]. As shown in Fig. 5, the overall pattern of GFAP immunoreactivity in the hippocampus suggested that the very high-fat diet (HF60), but not the HF45 diet, slightly promoted (*p* = 0.1) astrocyte hyperplasia, which is indicative of gliosis, a common response occurring in many types of brain injuries [46]. Only the hippocampi from 6 out of the 10 animals in the HF60 group were analysed, because the remaining ones were either not properly oriented or damaged. An increase in the number of animals analysed could have improved statistical significance.

### Correlation analysis between gene expression in the hippocampus or PBMC and anthropometric and circulating parameters

In order to further study the possible relationships between features of MONW-like animals and the expression of the genes studied in the hippocampus and PBMC, a Pearson’s correlation analysis was performed. The most remarkable correlations were observed with the HOMA index, the parameter that determines subjects’ insulin resistance. As expected, the HOMA index correlated positively with adiposity index, in both the HF45 and HF60 groups (*R* = 0.59; *p* = 0.01 and *R* = 0.56; *p* = 0.03, respectively). Moreover, in the hippocampus of the HF60 group the HOMA index was observed to correlate negatively with mRNA expression of *Naa16, Syn1, Tmcc2, Trkb* and *Zpr1*; and positively with the mRNA levels of *App* (Table 3). When considering the HF45 group, a negative correlation was found between the HOMA index and the mRNA expression of *Bdnf*, *Naa16*, *Tmcc2*, and *Zpr1* (Table 3). When analysing PBMC obtained at the age of six months, the most outstanding correlations were those obtained for *Syn1*, which correlated negatively with fasting glucose levels in the HF45 group and with insulin levels and the HOMA index in the HF60 group (Table 4). Remarkably, at the age of three months, after only one month of dietary intervention, *Syn1* gene expression in PBMC correlated negatively with circulating glucose and insulin levels, with HOMA-IR, and with visceral and subcutaneous fat in the HF60 group; and with circulating insulin, HOMA-IR, and visceral fat in the HF45 group (Table 4).

**Table 3:** Correlations between gene expression in the hippocampus and HOMA-IR at the age of 6 months

	HF45		HF60	
Genes	R	*p* value	R	*p* value
*App*	0.036	0.895	0.595*	0.032
*Bdnf*	−0.536*	0.027	−0.429	0.111
*Casp3*	−0.181	0.486	0.080	0.786
*Creb*	0.066	0.800	−0.248	0.372
*Fdnc5*	−0.403	0.137	0.337	0.260
*Naat16*	−0.507*	0.038	−0.622*	0.013
*Nrf2*	0.292	0.273	−0.172	0.541
*Pgc1α*	−0.207	0.460	0.383	0.196
*Sorl1*	−0.169	0.516	−0.489	0.064
*Syn1*	−0.312	0.223	−0.516*	0.049
*Tmcc2*	−0.535*	0.027	−0.738**	0.002
*Tnfα*	−0.159	0.604	0.561	0.091
*Trkb*	0.329	0.214	−0.769**	0.001
*Zpr1*	−0.533*	0.033	−0.693**	0.006

R Pearson correlation coefficients; boldface indicates statistical significance; *p < 0.05; **p < 0.01

**Table 4:** Correlations between *Syn1* gene expression in PBMC with fat and key metabolic parameters at the age of 3 and 6 months

	HF45		HF60	
Age of 3 months	R	*p* value	R	*p* value
Glucose	−0.349	0.358	−0.697*	0.017
Insulin	−0.850**	0.002	−0.697*	0.017
HOMA-IR	−0.791**	0.006	−0.755**	0.007
Visceral fat	−0.381	0.248	−0.707*	0.010
Subcutaneous fat	−0.623*	0.041	−0.682*	0.015
Age of 6 months	R	*p*	R	*p*
Glucose	−0.519*	0.047	−0.440	0.152
Insulin	−0.232	0.388	−0.561*	0.033
HOMA-IR	−0.235	0.380	−0.571*	0.033
Visceral fat	−0.125	0.621	−0.496	0.060
Subcutaneous fat	−0.309	0.213	−0.379	0.164

*R* Pearson correlation coefficients; boldface indicates statistical significance; *p < 0.05; **p < 0.01

## Discussion

It is well known that obesity and/or high-fat diet intake are associated with an increased risk of a wide range of metabolic disorders, such as type 2 diabetes, hypertension, stroke, or some types of cancer. More recently, the relationship between obesity and/or high-fat diet intake and cognitive decline has received some attention. In this study the aim was to evaluate whether the intake of unbalanced diets rich in fat administered in isocaloric amounts compared to a control diet and, thus, not promoting obesity, might be deleterious in terms of cognitive function when offered chronically. Moreover, we decided to identify early biomarkers of cognitive decline in PBMC, which have emerged as valuable for the detection of nutritional imbalance and markers of obesity-associated disorders [33, 34, 41].

Here, evidence is presented not only that excessive caloric intake (i.e. leading to obesity) is related to cognitive dysfunction, but our pair-fed study also shows that fat intake per se is an important harmful factor to be considered. Therefore, individuals with fat-rich unbalanced diets that do not develop obesity, those presenting the MONW phenotype, could potentially be a population at risk of developing not only the better known obesity-related complications, but also cognitive impairment and/or dementia in later life. The underlying mechanisms by which high-fat diet intake is related to cognitive decline are still not fully understood, although systemic and central inflammation has been suggested to be one of the most important pathophysiological mechanisms underlying cognitive impairment and dementia [7, 47]. This is because central inflammation could trigger a number of processes including oxidative stress and neuronal apoptosis [47–49]. Hence, in our study, the focus was on analysing genes associated with a particular cognitive decline-related process, and also on a pool of genes reflecting different, yet entwined, processes, i.e.: amyloid peptide metabolism, neuronal viability/function, synaptic function/signalling, transcription factor-mediated regulation of cognitive-related processes, and inflammation. Therefore, some of the genes have a direct role in modulating cognitive function while others have an indirect impact on such processes, e.g. reflecting central inflammation. All the genes analysed were selected based on data from microarray analysis conducted by our group, or from the literature where their expression changes in response to high fat diets or obesity were related to cognitive dysfunction.

Herein, the intake of a HF diet with 60% kcal from fats induced a clear MONW profile (i.e. greater visceral fat, fat depot in liver, and insulin resistance) in Wistar male rats. In these animals, increased mRNA expression of inflammatory (*Tnfα*) and pro-apoptotic (*Casp3*) markers was observed in hippocampus, even in the absence of increased body weight. Moreover, a tendency to astrogliosis was observed in the HF60 pair-fed animals, as seen in other studies using ad libitum HF diets [25, 50]. This is of relevance, as gliosis is a common characteristic of central nervous system damage and neurological diseases that include cognitive impairment [46, 51]. Further, mRNA expression of *App*, which codes for the precursor of the amyloid protein (APP) whose accumulation is an indicator of AD-like pathology, was also enhanced in the HF60 group, as described for HF diets administered ad libitum [50]. The enhancement of *App* in itself may not only involve a greater chance of subsequent amyloid deposition but may also exert pro-inflammatory effects [50] and enhance oxidative stress and apoptosis [52], thus promoting a vicious cycle of neuronal dysfunction. The decreased expression seen for *Sorl1* in this group, observed at protein level in both the HF45 and HF60 groups, is worthy of highlighting. To our knowledge, this is the first observation showing that the levels of *Sorl1* mRNA and protein in the hippocampus are associated with HF diet intake. Sortilin acts as a regulatory gate keeper for the trafficking pathways of *App* [53], protecting the latter from β secretase activity and thus preventing amyloidogenic peptide production and consequent deposition [54], a typical AD feature. Therefore, in addition to the increase in *App* mRNA levels in HF60 fed animals, the reduced mRNA and protein expression of sortilin in the hippocampus might worsen cognitive deficits. Isocaloric administration of a more moderate high-fat diet (45% kcal from fats) was not able to elicit these gene expression changes so explicitly as the very high-fat diet, although the same trends were detected. Nevertheless, the HF45 group displayed alterations in the expression of other interesting genes related to cognitive function, even without presenting MONW-related abnormalities as clearly as HF60 animals. For example, the expression of the gene coding for the auxiliary subunit of NatA (*Naa16*) was reduced in the hippocampus of animals maintained on both unbalanced diets. NatA is a protein complex that is believed to be involved in the regulation of protein turnover [55]. Asaumi et al. [56] demonstrated that, in humans, this complex interacts with APP and inhibits the secretion of amyloidogenic peptides. Here evidence is provided that the expression of the auxiliary subunit of NatA in rat hippocampus is modulated by HF diets and, based on the results from Asaumi et al., its down-regulation may contribute, to some extent, to cognitive decline in these animals. The same down-regulation pattern of expression was seen for *Zpr1* and *Tmcc2* in animals fed the HF diets. As stated by Nogusa et al [57], *Zpr1* may be related to oxidative stress. They reported that *Zpr1* is up-regulated in the hippocampus of mice fed a diet rich in saturated fat, which is in contrast with our results. Nevertheless, *Zpr1* is required for cell viability and contributes to normal cellular proliferation [58]. Thus, the inhibition of its expression could be associated with neuronal loss and subsequent cognitive deterioration. Little is known about the possible association between HF diets and the expression of *Tmcc2*, but decreased expression of both TMCC2 mRNA and protein was observed in our MONW-like animals. This gene codes for a protein that interacts with APP and could have a potential contribution to the anomalous beta-amyloid metabolism that occurs in AD; in fact, disruption of the gene *Tmcc2* has been proposed as a marker of AD [59, 60].

Obesity and ad libitum intake of HF diets have been related to a decrease in neuronal plasticity [61], which has been attributed to a variety of processes including abnormalities in *Bdnf* expression [62]. BDNF is a critical mediator of neuronal vitality and function, and has been revealed as a crucial factor in neuronal processes underlying learning and memory [63]. Although isocaloric intake of the high-fat diets did not alter *Bdnf* expression, the expression of down-stream effectors of BDNF action was reduced in the hippocampus of these animals, including *Trkb*, coding for BDNF receptor, and *Syn1,* which codes for an important protein for neurotransmitter release [64] and synaptic function [65]. The reduced mRNA expression of the latter is remarkable as it has been previously related to synaptic dysfunction in animals fed a high-fat, refined-sugar diet [61]; however, it was not accompanied by lower protein levels in our animals.

One of the characteristic features of the MONW phenotype is insulin resistance, which can be experimentally assessed using the HOMA index. Insulin resistance, a hallmark of obesity and of the intake of unbalanced diets, seems to play an important role in the modulation of gene expression observed in the hippocampus. Pearson’s correlation analysis shows that the degree of insulin resistance correlates negatively with the expression of *Bdnf*, *Naat16*, *Syn1*, *Tmcc2*, *Trkb,* and *Zpr1*, and positively with *App* mRNA levels when considering the HF60 group; and negatively with *Bdnf*, *Naat16*, *Tmcc2*, and *Zpr1* when considering the HF45 group. As a matter of fact, peripheral insulin resistance has been related to cognitive deficits in both humans [66, 67] and rodents [68]. Insulin receptors are distributed across the brain, particularly in the hippocampus and in other areas related to cognitive processing, where insulin signalling through its receptors is involved in synaptic plasticity and behaviour, proving it is a key component of hippocampal memory processes [16]. Arnold et al. [69] reported that central insulin resistance as a result of HF diet intake is associated with a decreased expression of synaptic markers in the hippocampus, suggesting that insulin acts as a synaptic modulator. Moreover, insulin is known to modulate Aβ processing within the brain, competing with this protein for degradation, and regulating its removal [70]. Thus, our results are in line with the above, suggesting that insulin plays a critical role in regulating cognitive processes; therefore, the impairment of its modulation would have deleterious effects on such processes.

Taken together, these molecular changes in the hippocampus suggest cognitive impairment in MONW-like animals that could eventually lead to neurodegenerative diseases. This was emphasised by our behavioural data, which demonstrates impaired spontaneous alternation in a T-maze after HF60 pair-feeding, thus providing additional support for hippocampal-based memory dysfunction resulting from isocaloric HF diet intake. These results are in agreement with studies on ad libitum administration of HF diets causing obesity, showing that cognitive performance is impaired when assessed using this or other spatial and non-spatial memory tasks [25, 71, 72].

The diagnosis of AD or dementia requires invasive and expensive techniques. Moreover, dementia is diagnosed when the disease is already present and sometimes fully developed. For these reasons, there is an imminent urgency to find early and valid biomarkers to detect cognitive disruption before dementia shows up. Based on our expertise in previous studies searching for the suitability of PBMC as a biological source of material to detect biomarkers of metabolic and/or homeostatic alterations after nutritional imbalances [32, 33, 41], we hypothesised that PBMC could be a suitable source of biomarkers of fat-induced neurodegenerative disease. PBMC are easily obtainable material that expresses 80% of the genome [73]. Moreover, these cells are exposed to different metabolites secreted by the central nervous system [74, 75]. It is known that about 500 ml of cerebrospinal fluid is taken up by blood every day [74]. Furthermore, dysfunction of the blood-brain barrier, an early pathologic feature of AD and vascular dementia, may enhance the exchange of metabolites between brain and blood in both directions [75]. Thus, PBMC could reflect gene expression changes occurring at a central level. In fact, it has previously been shown that PBMC can accurately reflect the gene expression pattern of orexigenic NPY which occurs in the hypothalamus in response to fasting-refeeding [32]. Here, our data show that PBMC express genes involved in cognitive function, and that their expression is regulated at different time points during rat development by chronic exposure to isocaloric HF intake in a similar manner to what is expected and observed in the hippocampus. Some of the gene expression changes were especially reflected at an early age in PBMC from animals maintained on both diets; this is the case of *Sorl1*, *Syn1*, and *Tmcc2*. Other genes, such as *Nrf2* and *Naa16*, displayed changes in their expression at later stages (five and six months of age) in response to both diets. It is worth mentioning that *Sorl1* and *Syn1* were revealed as potential early biomarkers of cognitive impairment, since their mRNA levels in PBMC remained significantly low with the intake of the HF diets throughout the dietary intervention. These results are in accordance with those observed in the hippocampus, and seem to be indicative of the disruption of cognitive processing that occurs at a central level after chronic exposure to HF diets even in the absence of obesity. Moreover, PBMC seem to be especially sensitive to nutritional imbalance, as although HF45 animals do not yet display changes in *Syn1* expression in the hippocampus they do show reduced mRNA levels of this gene in PBMC at different ages comparable to those of HF60 animals. *Syn1* could be a particularly interesting biomarker of cognitive impairment associated with the intake of HF diets in PBMC, as its expression in these cells has a strong negative correlation with insulin resistance-related parameters. More research is needed, either in rodents or directly in humans, to confirm the usefulness of *Syn1* and to validate it as an early marker of diet-related cognitive impairment.

## Conclusion

Continuous intake of unbalanced diets with an excess of fat has a great impact on the expression of cognitive-related genes in the hippocampus, suggesting cognitive impairment in these animals. To our knowledge, this is the first time a link has been established between the MONW phenotype and cognitive dysfunction. Moreover, PBMC are able to reflect impairment in the expression of cognitive-related genes observed in the hippocampus. Although further studies are needed, considering that neither MONW-like individuals nor dementia are easily diagnosed, and the lack of effective treatments against the latter, PBMC open the possibility to perform human studies with minimum invasion, since they could be a promising approach for the identification of accessible early predictors that reflect cognitive impairment before any other pathological signs appear.

## Additional file

Additional file 1: Nucleotide sequences of primers used for real-time RT-qPCR amplification. (DOCX 17 kb)

## Abbreviations

AD: Alzheimer’s disease
*App*: Amyloid precursor protein
*Bdnf*: Brain derived neurotrophic factor
*Casp3*: Caspase 3
*Creb*: cAMP responsive element binding protein 1
CVD: Cardiovascular disease
ELISA: Enzyme-linked immunosorbent assay
*Fndc5*: Fibronectin type III domain containing 5
*Gdi1*: GDP dissociation inhibitor 1
GFAP: Glial fibrillary acidic protein
HF: High fat
*Itgβ1*: Integrin β1 subunit
MCI: Mild cognitive impairment
MONW: Metabolically obese, normal-weight
*Naa16*: N(alpha)-acetyltransferase 16, NatA auxiliary subunit
*Nrf2*: NF-E2-related factor 2
PBMC: Peripheral blood mononuclear cells
*Pgc1α*: PPARG coactivator 1 alpha
*Rplp0*: Ribosomal protein lateral stalk subunit P0
RT-qPCR: Real-time reverse transcription polymerase chain reaction
*Sorl1*: Sortilin related receptor 1
*Syn1*: Synapsin I
*Tmcc2*: Transmembrane and coiled-coil domain family 2
*Tnfα*: Tumour necrosis factor α
*Trkb*: Neurotrophic receptor tyrosine kinase 2
*Zpr1*: Zinc finger protein 259
