# Supplementary material for: Cognitive impairment in metabolically-obese, normal-weight rats: identification of early biomarkers in peripheral blood mononuclear cells
Source: Mol Neurodegener. 2018 Mar 22;13:14. doi: 10.1186/s13024-018-0246-8 (PMC5863821; doi:10.1186/s13024-018-0246-8)
Supplement: Supplementary file 1 — Nucleotide sequences of primers used for real-time RT-qPCR amplification. (DOCX 17 kb) [file 13024_2018_246_MOESM1_ESM.docx]

| Gene | Forward primer (5’-3’) | Reverse primer (3’-5’) | Amplicon size (bp) |
| --- | --- | --- | --- |
| *App* | CTGCTGACCGAGGACTGAC | CCGAACTCCGCATCCATCTT | 94 |
| *Bdnf* | ATTAGCGAGTGGGTCACAGC | CGAGTTCCAGTGCCTTTTGT | 189 |
| *Casp3* | ACCCTGAAATGGGCTTGTGT | ACAGGTCCGTTCGTTCCAAA | 280 |
| *Creb* | CAGGGAGGAGCAATACAGC | GGAGGACGCCATAACAACT | 255 |
| *Fndc5* | atgaaggagatggggaggaa | Gcggcagaagagagctataaca | 102 |
| *Naat16* | AGTGCTACCGAAATGCCCTCA | TGTGTCGGGCGTAACTGAAGA | 139 |
| *Nrf2* | CAGAGCAAGTGACGAGATGG | CCGAAATGTTGAGTGTGGTG | 177 |
| *Pgc1α* | CATTTGATGCACTGACAGATGGA | CCGTCAGGCATGGAGGAA | 70 |
| *Sorl1* | caccgtctcattgtcagcac | atctcgtagcccctggtttc | 123 |
| *Syn1* | GCAGTTTGGTCATTGGGCTG | ACAGGGTATGTTGTGCTGCT | 202 |
| *Tmcc2* | TCCTCCTCTACCACCGATACC | CTCCCTTGTCACCCTTGTCC | 103 |
| *Tnf α* | CCGATTTGCCATTTCATACC | TCGCTTCACAGAGCAATGAC | 237 |
| *Trkb* | TCGGTATCACCAACAGCCAG | GCTCGGGGCAGAGGTTATAG | 141 |
| *Zpr1* | GGATTACCCTCCACATCACAG | TGTCTTTCAGCAGTCCTTCG | 153 |

**Additional file 1.** Nucleotide sequences of primers used for real-time RT-qPCR amplification.

Abbreviations: *App*, amyloid precursor protein; *Bdnf*, brain derived neurotrophic factor; *Casp3*, caspase 3; *Creb*, cAMP responsive element binding protein 1; *Fndc5*, fibronectin type III domain containing 5; *Naa16*, N(alpha)-acetyltransferase 16, NatA auxiliary subunit; *Nrf2*, NF-E2-related factor 2; *Pgc1α,* PPARG coactivator 1 alpha; *Sorl1*, sortilin related receptor 1; *Syn1*, Synapsin I; *Tmcc2*, transmembrane and coiled-coil domain family 2; *Tnfα,* tumour necrosis factor α; *Trkb*, neurotrophic receptor tyrosine kinase 2; *Zpr1*, zinc finger protein 259.
